# Structural instability of large-scale functional networks

**DOI:** 10.1371/journal.pone.0181247

**Published:** 2017-07-20

**Authors:** Shogo Mizutaka, Kousuke Yakubo

**Affiliations:** 1 School of Statistical Thinking, The Institute of Statistical Mathematics, Tachikawa, Tokyo, Japan; 2 Department of Applied Physics, Hokkaido University, Sapporo, Hokkaido, Japan; Universite de Namur, BELGIUM

## Abstract

We study how large functional networks can grow stably under possible cascading overload failures and evaluated the maximum stable network size above which even a small-scale failure would cause a fatal breakdown of the network. Employing a model of cascading failures induced by temporally fluctuating loads, the maximum stable size *n*_max_ has been calculated as a function of the load reduction parameter *r* that characterizes how quickly the total load is reduced during the cascade. If we reduce the total load sufficiently fast (*r* ≥ *r*_c_), the network can grow infinitely. Otherwise, *n*_max_ is finite and increases with *r*. For a fixed *r*(< *r*_c_), *n*_max_ for a scale-free network is larger than that for an exponential network with the same average degree. We also discuss how one detects and avoids the crisis of a fatal breakdown of the network from the relation between the sizes of the initial network and the largest component after an ordinarily occurring cascading failure.

## Introduction

Numerous complex systems in nature and society can be simplified and abstracted by describing them as networks, in which nodes and edges represent constituent elements and their interactions, respectively. Extensive studies [1–3] have revealed common statistical features of real-world complex networks, such as the small-world property [4], the scale-free property [5], community structures [6], and degree-degree correlations [7]. In order to clarify the origin of these features and/or properties of various dynamics on such networks, so many network models have been proposed so far. In most of previous network models, the number of nodes *N*, namely the network size, is treated as an a priori given parameter. The network size can then take any value, and, as is often the case, the limit of infinite *N* is taken in order to simplify the analysis. Thus, these models implicitly assumes that networks are stably present no matter how large networks grow. This assumption is, however, not always valid in real-world systems. In an ecological network representing a closed ecosystem, for example, too many species destabilize the ecosystem and the number of species (nodes in the ecological network) cannot increase unboundedly [8, 9]. Also in a trading network, too many firms make the network fragile because of unstable cartels [10], increase of financial complexity [11, 12], possible large-scale chain-bankruptcy, and other risks [13]. As in these examples, due to intrinsic instability of large-scale networks, some sort of networks have their own limit in sizes only below which they can be stable [14, 15].

It is important to study limit sizes of connected networks and find a way to control them. Such information for *functional networks* is particularly crucial, because unstable functional networks are directly connected to the instability of our modern society supported by them. Functions provided by functional networks are guaranteed by global connectivity and normal operation of each node (or edge). However, the larger a network grows, the lower the probability that all the nodes operate normally becomes. When failures are caused by *overloads*, even a few failures can spread to the entire network through a cascading process, which leads the fatal breakdown of the network [16–21]. It is then significant to investigate how large functional networks can grow, while maintaining its global connectivity, by overcoming cascading overload failures. In this paper, we evaluate the upper limit of connected network size above which the network becomes unstable by cascading overload failures and discuss how the maximum stable size can be controlled. We examine uncorrelated random networks with Poisson and power-law degree distributions by employing the model of cascading failures triggered by temporally fluctuating loads [22].

## Model and methodology

### Model

In this section, we outline the model of cascading failures induced by temporally fluctuating loads [22]. In functional networks such as power grids, the Internet, or trading networks, some sort of “flow” (electric current in a power grid, packet flow in the Internet, and money flow in a trading network) realizes their functions. And flow, at the same time, plays a role of “loads” in these networks. The load on a node usually fluctuates temporally and the node fails if the instantaneous value of the load exceeds the node capacity. Since flux fluctuations at a node exhibit the same scaling behavior with fluctuations of the number of non-interacting random walkers on the node [23, 24], Kishore *et al.* modeled fluctuating loads by random walkers moving on a network and calculated the overload probability that the number of walkers exceeds the range allowed for a node [25]. The model of cascading failures employed in the present work utilizes this overload probability.

In a connected and undirected network with *M*_0_ edges, the probability *h*_*k*_(*w*) that *w* random walkers (loads) exist on a node of degree *k* is presented by

hk(w)=(W0w)pkw(1-pk)W0-w, (1)

where *W*_0_ is the total number of walkers and *p*_*k*_ = *k*/2*M*_0_ is the stationary probability to find a random walker on a node of degree *k* [26]. This leads a natural definition of the capacity *q*_*k*_ as

$$\begin{array}{c} q_{k}={\left\langle w\right\rangle }_{k}+m\sigma_{k}, \end{array}$$ (2)

where *m* is a real positive parameter characterizing the node tolerance, and 〈*w*〉_*k*_ and *σ*_*k*_ are the average and the standard deviation of *h*_*k*_(*w*), which are given by 〈*w*〉_*k*_ = *W*_0_*p*_*k*_ and $\sigma_{k}=\sqrt{W_{0}p_{k}\left(1-p_{k}\right)}$, respectively. Since the overload probability $F_{W_{0}}\left(k\right)$ is the probability of *w* to exceed *q*_*k*_, we have [25]

FW0(k)=∑w=⌊qk⌋+1W0(W0w)pkw(1-pk)W0-w=Ik/2M0(⌊qk⌋+1,W0-⌊qk⌋), (3)

where *I*_*p*_(*a*, *b*) is the regularized incomplete beta function [27] and ⌊*x*⌋ denotes the largest integer not greater than *x*.

Using the above overload probability, the cascade process of overload failures is defined as follows [22]:

(i) Prepare an initial connected, uncorrelated, and undirected network $G_{0}$ with *N* nodes and *M*_0_ edges, in which totally *W*_0_ random walkers exist, and determine the capacity *q*_*k*_ of each node according to Eq (2). *W*_0_ is set as *W*_0_ = *aM*_0_, where the parameter *a* is the load carried by a single edge.
(ii) At each cascade step *τ*, reassign *W*_*τ*_ walkers to the network $G_{\tau }$ at step *τ*, where the total load *W*_*τ*_ is given by
  $$\begin{array}{c} W_{\tau }={\left(\frac{M_{\tau }}{M_{0}}\right)}^{r}W_{0}\;. \end{array}$$ (4)
  Here, *M*_*τ*_ is the total number of edges in the network $G_{\tau }$ and *r* is a real positive parameter.
(iii) For every node in $G_{\tau }$, calculate the overload probability given by
  $$\begin{array}{c} F_{W_{\tau }}\left(k_{0},k\right)=I_{k/2M_{\tau }}(\left\lfloor q_{k_{0}}\left(W_{0}\right)\right\rfloor +1,W_{\tau }-\left\lfloor q_{k_{0}}\left(W_{0}\right)\right\rfloor ), \end{array}$$ (5)
  where *k*_0_ and *k* are the initial degree and the degree of the node at cascade step *τ*, and remove nodes from $G_{\tau }$ with this probability.
(iv) Repeat (ii) and (iii) until no node is removed in the procedure (iii).

The reduction of the total load in the procedure (ii) corresponds to realistic situations in which the total load is reduced to some extent during a cascade process to prevent the fatal breakdown of the network function. When a company goes bankrupt on a trading network, for example, a large-scale chain bankruptcy would be prevented by the reduction of the total debt (loads) realized by financial bailout measures. The exponent *r* characterizes how quickly the total load decreases with decreasing the network size, which is called the load reduction parameter.

During the cascade, the network $G_{\tau }$ might be disconnected even though the initial network $G_{0}$ is connected. In such a case, a walker on a connected component cannot jump to other components. Therefore, the amount of walkers on each component is conserved in the random walk process. The overload probability then becomes dependent on how the total load is distributed to disconnected components. Thus the overload probability deviates from Eq (5). This deviation is, however, small and the effect of disconnected components can be approximately neglected as argued in details in Ref. [22]. The validity of this approximation will be confirmed in the next section by numerical simulations in which walkers distributed proportionally to the number of edges in each component cannot move to other components.

### Size of the largest component

We examine the stability of a network under cascading overload failures described above by analyzing the size *n*_f_ of the largest connected component in the network after completed the cascading process. If *n*_f_ is very small, the initial network is considered to be unstable. The quantity *n*_f_ obviously depends on the initial network size *N*, and the maximum value *n*_max_ of *n*_f_ with respect to *N* provides the upper limit of the size of stable connected networks in a given cascading condition. The surviving component of size *n*_max_ may experience further cascading failures after a long time, but simultaneously the component can grow during this period. In the competition between the growth and decay processes, the component smaller than *n*_max_ can, in substance, stably grow up to *n*_max_. Therefore, the connected network size fluctuates around this maximum size *n*_max_.

In order to calculate the maximum stable size *n*_f_, we construct a master equation for the probability *Π*_*τ*_(*k*_0_, *k*) that a randomly chosen node has the degree *k* at cascade step *τ* and the initial degree *k*_0_. It is convenient to introduce another probability *ϕ*_*τ*_(*k*) of a node adjacent to a randomly chosen node of degree *k* to experience an overload failure at cascade step *τ*. This probability is independent of *k* for uncorrelated networks and is given by [22]

$$\begin{array}{c} \phi_{\tau }=\sum_{k_{0}}\sum_{k^{\prime }=1}^{k_{0}}\frac{k^{\prime }\Pi_{\tau }\left(k_{0},k^{\prime }\right)}{{\left\langle k\right\rangle }_{\tau }}F_{W_{\tau }}\left(k_{0},k^{\prime }\right), \end{array}$$ (6)

where 〈*k*〉_*τ*_ is the average degree of $G_{\tau }$. We then formulate the master equation for Π_*τ*_(*k*_0_, *k*) as

Πτ(k0,k)=∑k′≥kΠτ-1(k0,k′){(k′k)ϕτ-1k′-k(1-ϕτ-1)k[1-FWτ-1(k0,k′)] + δk0FWτ-1(k0,k′)}. (7)

In this equation, we do not remove overloaded nodes actually but leave them in the system as zero-degree nodes, which makes the theoretical treatment easier. The right-hand side of this equation represents the probability that a degree-*k*′ node in $G_{\tau -1}$ becomes a node of degree *k* at cascade step *τ*. The first term describes the situation that the degree-*k*′ node does not experience an overload failure and *k*′ − *k* nodes adjacent to this node fail. The second term stands for the case that the degree-*k*′ node itself fails and becomes a zero-degree node. Solving numerically Eq (7) with the aid of Eq (6), we can calculate Π_*τ*_(*k*_0_, *k*) iteratively starting from $\Pi_{0}\left(k_{0},k\right)=P_{0}\left(k\right)\delta_{kk_{0}}$, where *P*_0_(*k*) is the degree distribution function of $G_{0}$. According to the procedure (iv), we stop this iterative calculation at step $\tilde{\tau }$ satisfying the condition

$$\begin{array}{c} \sum_{k,k_{0}}F_{W_{\tilde{\tau }}}\left(k_{0},k\right)\Pi_{\tilde{\tau }}\left(k_{0},k\right)<\frac{1}{N}, \end{array}$$ (8)

which implies that the expectation number of overloaded nodes becomes less than unity.

We can obtain the largest connected component size *n*_f_ at the final cascade step $\tilde{\tau }$ from the degree distribution $P_{\tilde{\tau }}\left(k\right)$ of $G_{\tilde{\tau }}$ which is given by $P_{\tilde{\tau }}\left(k\right)=\sum_{k_{0}\geq k}\Pi_{\tilde{\tau }}\left(k_{0},k\right)$. Employing the generating function formalism, *n*_f_ is calculated by [28]

$$\begin{array}{c} n_{\text{f}}=N[1-\sum_{k}P_{\tilde{\tau }}\left(k\right)u^{k}], \end{array}$$ (9)

where *u* is the smallest non-negative solution of

$$\begin{array}{c} u=G_{1}\left(u\right), \end{array}$$ (10)

and *G*_1_(*x*) is the generating function of the remaining degree distribution, which is defined by

$$\begin{array}{c} G_{1}\left(x\right)=\frac{1}{{\left\langle k\right\rangle }_{\tau }}\sum_{k}\left(k+1\right)P_{\tilde{\tau }}\left(k+1\right)x^{k}. \end{array}$$ (11)

It should be noted that Eq (9) does not mean that *n*_f_ is proportional to *N* because $1-\sum_{k}P_{\tilde{\tau }}\left(k\right)u^{k}$ depends on *N*.

## Results

First, we calculated *n*_f_ for the Erdős-Rényi random graph (ERRG) as an initial network $G_{0}$. In this case, the binomial degree distribution function for $G_{0}$ is given by

**Fig 1:**
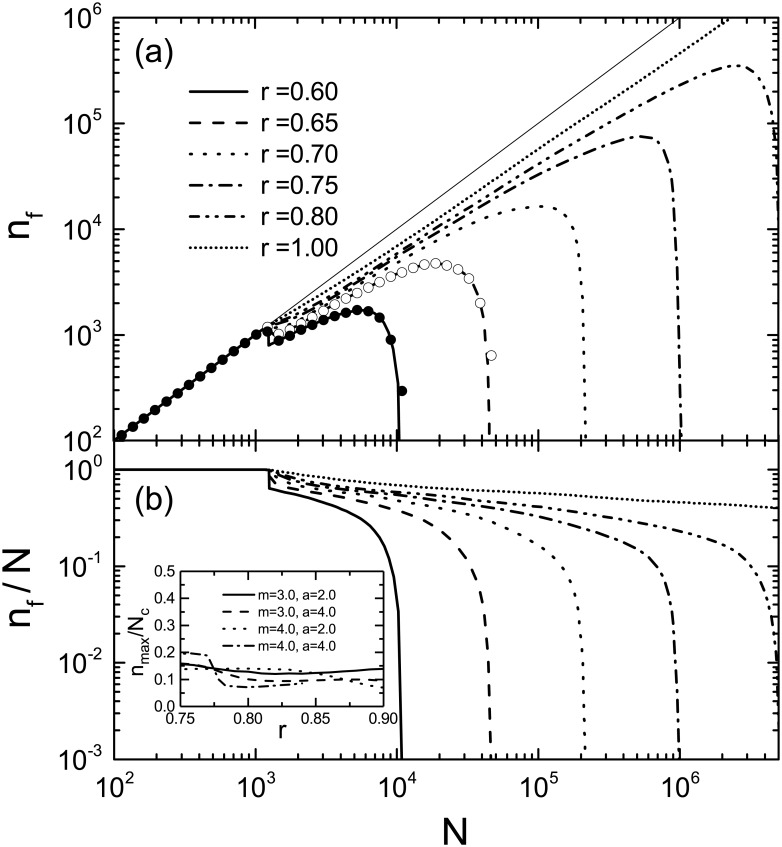
Relation between the largest component size *n*_f_ and the initial network size *N*. (a) Largest component size *n*_f_ after completing cascading overload failures as a function of the size *N* of initial ERRGs, for various values of *r*. The thick lines indicate *n*_f_ calculated by Eq (9) and filled and open circles on thick lines show the results obtained by the numerical simulation described in the main text. The thin straight line is a guide to the eyes for *n*_f_ = *N*. (b) Relative largest component size *n*_f_/*N* as a function of *N*. Lines have the same meanings as those in (a). All the results are calculated for 〈*k*〉_0_ = 5.0, *m* = 4.0, and *a* = 2.0. The inset shows the *r* dependence of *n*_max_/*N*_c_. Lines in the inset represent results for ERRGs with different values *m* and *a*.

P0(k)=(N-1k)pk(1-p)N-k-1, (12)

where *p* = 〈*k*〉_0_/*N*. In this work, we fix the initial average degree as 〈*k*〉_0_ = 5.0. Although initial networks having this average degree are not completely connected with isolated nodes at a very low rate, this does not affect our conclusion. Fig 1 shows *n*_f_ as a function of the initial network size *N* for various values of the load reduction parameter *r*. For these results, the node tolerance parameter *m* and the load carried by a single edge, *a*, are chosen as *m* = 4.0 and *a* = 2.0. The lines in Fig 1 represent *n*_f_ calculated by Eq (9) and the symbols indicate the results obtained by numerical simulations performing faithfully the cascade process from (i) to (iv) described in the Model section. In the numerical simulation, the overload probability at cascade step *τ* is calculated under the condition that random walkers cannot jump to other components. Namely, instead of Eq (5), we adopt the overload probability of a node in the *α*-th component given by

$$\begin{array}{c} F_{W_{\tau }^{\alpha }}\left(k_{0},k\right)=I_{k/2M_{\tau }^{\alpha }}(\left\lfloor q_{k_{0}}\left(W_{0}\right)\right\rfloor +1,W_{\tau }^{\alpha }-\left\lfloor q_{k_{0}}\left(W_{0}\right)\right\rfloor ), \end{array}$$ (13)

where $M_{\tau }^{\alpha }$ is the number of edges in the *α*-th component of $G_{\tau }$ and $W_{\tau }^{\alpha }=\left(M_{\tau }^{\alpha }/M_{\tau }\right)W_{\tau }$. The remarkable agreement between the symbols and the lines suggests that our approximation by Eq (5) is quite accurate.

The quantity *n*_f_ shown in Fig 1 is exactly equal to *N* as long as *N* < N* (≈ 10^3^), regardless of the value of *r*. This implies that the network never experiences overload failures until the network grows up to N*. Thus, N* is determined by

$$\begin{array}{c} N^{*}=\frac{1}{\sum_{k}F_{W_{0}}\left(k\right)P_{0}\left(k\right)}, \end{array}$$ (14)

which does not depend on *r*. When the network grows larger than N*, it starts to decay by initial failures and subsequent avalanche of failures. The largest component size *n*_f_ after the cascade then becomes smaller than *N*, but still increases with *N*, at least unless *N* is much larger than N*. For *r* ≤ 0.8, when *N* exceeds a certain value *N*_c_(*r*), *n*_f_ rapidly decreases with *N*. Therefore, *n*_f_ becomes maximum at *N* = *N*_c_. This maximum value of *n*_f_ is nothing but *n*_max_ mentioned at the beginning of the Size of the largest component section. Fig 1 clearly shows that the maximum stable size *n*_max_ is an increasing function of *r*. This is because a large value of *r*, namely a rapid decrease of the total load *W*_*τ*_ during the cascade, prevents large-scale cascading failures in our model.

**Fig 2:**
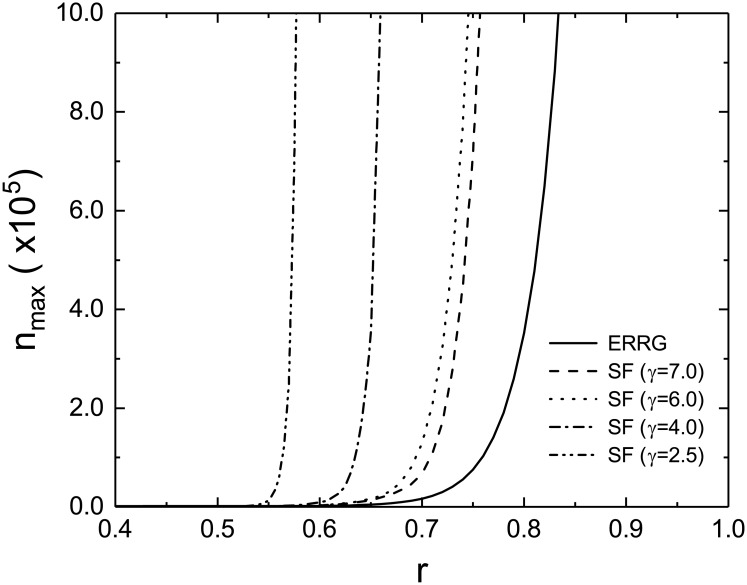
Maximum stable size *n*_max_ as a function of the load reduction parameter *r*. Three lines represent the results for the ERRG (solid line) and SF networks with the degree distributions *P*_0_(*k*) given by Eq (15) with *γ* = 7.0 (dashed line), 6.0 (dotted line), 4.0 (dashed-dotted line), and 2.5 (dashed-two dotted line). The minimum and maximum degrees *k*_min_ and *k*_max_ in Eq (15) are set as 1 and 80, respectively. All the results are calculated for 〈*k*〉_0_ = 5.0, *m* = 4.0, and *a* = 2.0.

The *r* dependence of *n*_max_ is closely related to the percolation transition by cascading overload failures. As pointed out by Ref. [22], there exists a critical value *r*_c_ above which the largest component size diverges in proportion to *N* in the thermodynamic limit. Thus, *n*_f_ goes to infinity as *N* → ∞ for *r* ≥ *r*_c_, which implies the absence (divergence) of *n*_max_. On the other hand, for *r* < *r*_c_, *n*_f_ is finite and varies with *N* to be maximized at *N*_c_ as mentioned above. As a consequence, *n*_max_ increases with *r* for *r* < *r*_c_ and diverges at *r* = *r*_c_. A finite-size scaling analysis [29] predicts that *n*_max_ for *r* < *r*_c_ behaves as $n_{\text{max}}\propto {\left|r-r_{\text{c}}\right|}^{\beta -\nu^{*}}$ if *r* is close enough to *r*_c_, where the correlation volume exponent ν* and the order parameter exponent *β* characterize *N*_c_ and *n*_max_/*N* as $N_{\text{c}}\propto {\left|r-r_{\text{c}}\right|}^{-\nu^{*}}$ for large enough *N*_c_ and *n*_max_/*N* ∝ (*r* − *r*_c_)^*β*^ for large enough *N*, respectively. Such a behavior is demonstrated by the solid line in Fig 2 for the ERRG. The result suggests that the load control during a cascade is crucial to realize large functional networks.

**Fig 3:**
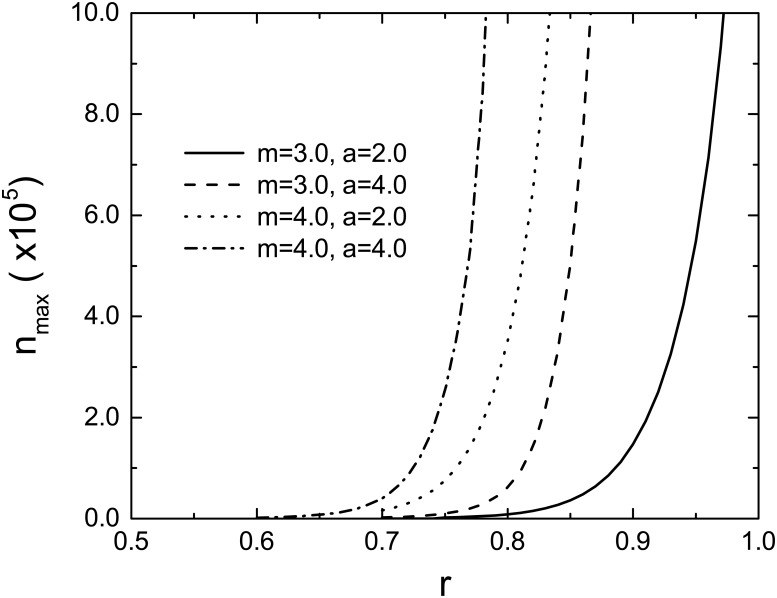
Maximum stable size *n*_max_ for the ERRG with 〈*k*〉_0_ = 5.0 as a function of the load reduction parameter *r*. Four lines represent the results for different combinations of the node tolerance parameter *m* and the load carried by a single edge *a*.

How is the maximum size *n*_max_ affected by properties of the initial network $G_{0}$? To clarify the influence of the degree inhomogeneity in $G_{0}$, we first calculate *n*_max_ for scale-free (SF) networks with the degree distribution given by

$$\begin{array}{c} P_{0}\left(k\right)=\{\begin{array}{cc} 0 & \text{if}\;k<k_{\text{min}}\;\text{or}\;k>k_{\text{max}}\\ \frac{c}{k^{\gamma }+d^{\gamma }} & \text{if}\;k_{\text{min}}\leq k\leq k_{\text{max}}, \end{array} \end{array}$$ (15)

where *d*, *γ*, *k*_min_, *k*_max_ and the normalization constant *c* are real positive constants. The degree distribution has asymptotically a power-law form, i.e., *P*(*k*) ∼ *k*^−*γ*^ for *k* ≫ *d*. The average degree 〈*k*〉_0_ can be controlled by *d* for a specific value of *γ*. The results for various scale-free networks are shown in Fig 2. The maximum stable sizes for the SF networks are obviously greater than that for the ERRG. This implies that an SF network is more stable than the ERRG with the same average degree, which is consistent with the previous result showing the robustness of SF networks against cascading overload failures [22]. Next, we calculate *n*_max_ for several combinations of the node tolerance parameter *m* and the load carried by a single edge *a*. The results depicted in Fig 3 clearly indicate that *n*_max_ increases with both *m* and *a*. It is obvious that the larger the node tolerance parameter *m*, the more stable the network consisting of tolerant nodes becomes. The monotonous increase of *n*_max_ with *a* is due to the fact that $F_{W_{\tau }}\left(k_{0},k\right)$ given by Eq (5) is a decreasing function of *a*.

It is interesting to notice that the maximum stable size *n*_max_ shown in Fig 1(a) is roughly proportional to *N*_c_. In fact, the ratio *n*_max_/*N*_c_ is about 0.1 to 0.15 independently of *r*, unless *r* is close to *r*_c_. Here we should note that this ratio varies with *N*_c_ according to $n_{\text{max}}/N_{\text{c}}\propto N_{\text{c}}^{-\beta /\nu^{*}}$ if *r* is close enough to *r*_c_. The ratio *n*_max_/*N*_c_ is also insensitive to the parameters *m* and *a*, as shown in the inset of Fig 1(b). Furthermore, as shown by Fig 1(b), the ratio *n*_f_/*N* slowly decreases with *N* if *n*_f_/*N* ≳ *n*_max_/*N*_c_ but turns to a rapid decrease when *n*_f_/*N* becomes less than *n*_max_/*N*_c_(∼ 0.1). These empirical facts give us significant information about the stability of the network. If the size of the largest component after cascading overload failures falls close to 10% to 15% of the size of the network before the cascade, the network is in immediate danger of a fatal breakdown. In order to accomplish further stable growth of the network, we need to raise the load reduction parameter *r*. Of course, the value of *n*_max_/*N*_c_ is peculiar to the present cascade model. However, it has been found that qualitative properties of our model are robust against changes in details of the model as long as failures are induced by temporally fluctuating loads [22]. Therefore, even for a real-world functional network, the ratio *n*_f_/*N* is supposed to decrease drastically with *N* when this ratio falls below a certain value. Our results suggest that we must take measures to prevent a fatal breakdown of a functional network if the decreasing rate of *n*_f_/*N* with increasing the network size becomes higher than its ordinary value.

## Conclusions

We have studied how large a functional network exposed to cascading overload failures can grow stably and evaluated the maximum stable size *n*_max_ above which the network would face the crisis of a fatal breakdown. To this end, we employed the model of cascading overload failures triggered by fluctuating loads [22] which is described by random walkers moving on the network [25]. In this model, how quickly the total load is reduced during the cascade to prevent the fatal breakdown is quantified by the load reduction parameter *r*. The maximum stable size *n*_max_ was calculated by using the generating function technique and solving the master equation for the probability *Π*_*τ*_(*k*_0_, *k*) that a randomly chosen node has the degree *k* at cascade step *τ* and the initial degree *k*_0_. Our results show that *n*_max_ is an increasing function of *r* and diverges at a certain value *r*_c_. This implies that the faster the total load is reduced during the cascade, the larger the network can grow, and we can realize an arbitrarily large network if the total load is sufficiently quickly reduced (*r* ≥ *r*_c_). It has been also clarified that the degree inhomogeneity improves stability of the network. More precisely, for a given *r*(< *r*_c_), *n*_max_ for a scale-free network is larger than that for the Erdős-Rényi random graph with the same average degree. Furthermore, from the empirical relation between *n*_max_ and the network size *N*_c_ giving *n*_max_, we argued how one detects and avoids the crisis of the network breakdown. The present results suggest that a certain relative size of the largest component after cascading failures could be a sign for the impending network collapse. For further stable growth of the network, a more rapid reduction of the total load is required during a cascade of overload failures.

In this paper, we investigated only uncorrelated networks, while most of real-world functional networks have correlations between nearest neighbor degrees. For a correlated network, the probability *ϕ*_*τ*_ of a node adjacent to a randomly chosen node of degree *k* to experience an overload failure at cascade step *τ* depends on *k*. This is in contrast to the case of uncorrelated networks, where *ϕ*_*τ*_ given by Eq (6) is independent of *k*. Thus, the analysis becomes much more complicated for correlated networks than the present study. However, we suppose that our conclusion does not change qualitatively though *n*_max_ depends on the strength of the degree correlation. A positive (negative) degree correlation simply makes a network robust (fragile) against various types of failures [30–34]. Thus, it seems plausible that also for our failure dynamics the degree correlation merely shifts the value of *n*_max_ upward or downward. Nevertheless, the relation between the stability of a functional network and its size must be strongly affected by the model of cascading failures. We hope that the problem of spontaneous instability in largely grown networks will be studied more extensively in diverse ways and models.
